# Robotic Segmental Resection of the Splenic Flexure and Mid-Transverse Colon for Malignancy Treatment: A Systematic Review of Operative Techniques, Anastomotic Approaches, and Surgical and Oncological Outcomes

**DOI:** 10.3390/jcm14207236

**Published:** 2025-10-14

**Authors:** Alessia Fassari, Angelo Iossa, Alessandra Micalizzi, Giulio Lelli, Sara Giovampietro, Edoardo Rosso, Giuseppe Cavallaro

**Affiliations:** 1 Centre Hospitalier de Luxembourg, L-1210 Luxembourg, Luxembourg; alessia.fassari@gmail.com; 2 Department of Medico-Surgical Sciences and Biotechnologies, Sapienza University, 00185 Rome, Italy; angelo.iossa@uniroma1.it (A.I.); alessandra.micalizzi@uniroma1.it (A.M.); giuliolelli1987@gmail.com (G.L.); saragiova91@gmail.com (S.G.); 3 Pôle Santé Sud, 72100 Le Mans, France; edoardo_rosso@hotmail.com; 4 Department of Surgery, Sapienza University, 00185 Rome, Italy

**Keywords:** robotic surgery, segmental colectomy, splenic flexure cancer, transverse colon cancer, minimally invasive surgery

## Abstract

**Background/Objectives:** The potential role of robotic surgery in segmental colectomy for the treatment of splenic flexure and mid-transverse colon cancers remains underexplored. These sites are technically demanding because of the occurrence of vascular variability, the need for dual lymphatic drainage, and the close anatomical relationship to surrounding organs. This systematic review evaluated surgical strategies, anastomotic techniques, perioperative outcomes, and the oncological adequacy of robotic segmental colectomies in this context. **Methods:** The review followed the PRISMA guidelines (PROSPERO ID: CRD420251119736). Studies were eligible if they included ≥3 patients who were undergoing a robotic segmental colectomy for malignant tumors of the splenic flexure or mid-transverse colon. Data on patient demographics, operative details, complications, and oncological outcomes were extracted. The risk of bias was assessed using the Newcastle–Ottawa Scale and ROBINS-I. **Results:** Five retrospective studies reporting on 74 patients were included. All the procedures involved a fully robotic approach. Vascular ligation was uniform for transverse tumors (middle colic vessels point of origin), but varied for splenic flexure lesions. Anastomotic reconstruction was extracorporeal stapled (55.4%), intracorporeal stapled (16.2%), or intracorporeal hand sewn (4.1%). Operative times were in the range of 157.5–268 min; conversion occurred in 4.1% of cases. The overall morbidity was 16.2%, with anastomotic leaks in 5.4% of cases. No 30-day mortality was observed, and one reoperation was required. All patients achieved R0 resection, with a mean lymph node yield of 16.9. Only one recurrence was documented during the follow-up period. **Conclusions:** Robotic segmental colectomy for splenic flexure and mid-transverse colon malignancies is feasible and safe, achieving consistent perioperative and oncological outcomes. Larger multicenter prospective studies are needed to validate the oncological adequacy, standardize anastomotic strategies, and assess the cost effectiveness of the approach.

## 1. Introduction

Oncologically, splenic flexure tumors, defined as those arising between the distal third of the transverse colon and within 10 cm of the descending colon, together with mid-transverse lesions, represent some of the most technically demanding sites for colorectal resection. Surgical complexity arises from the need to mobilize both colonic flexures, working across multiple abdominal quadrants, in close proximity to the spleen, pancreas, stomach, and left kidney. Adequate lymphadenectomy requires precise identification and skeletonization of the middle colic vessels, whose branching pattern and variability may complicate vascular control [1,2,3]. Moreover, patients are often diagnosed at an advanced stage of disease, because subocclusive symptoms are usually the first manifestation and indicate tumors that are already voluminous. Finally, there is no consensus in the literature regarding the optimal surgical strategy, with proponents advocating for extended right or left hemicolectomy versus more conservative segmental resections [4,5].

Robotic surgery may facilitate these complex resections by enabling precise dissection, secure vascular ligation, and improved intracorporeal reconstruction [6]. Nevertheless, current evidence on the perioperative outcomes and oncological safety of such procedures is fragmented, being limited to case reports and a small heterogeneous series of studies. In addition, the choice of anastomotic technique (manual vs. mechanical, intracorporeal vs. extracorporeal) could affect the complication rates and recovery, but data specific to this setting remain scarce [7].

The aim of this systematic review is to critically assess and synthesize the available evidence on robotic segmental colectomies for malignant tumors of the splenic flexure and mid-transverse colon, focusing on surgical strategies, anastomotic techniques, and perioperative and oncological outcomes.

## 2. Methods

**Figure 1:**
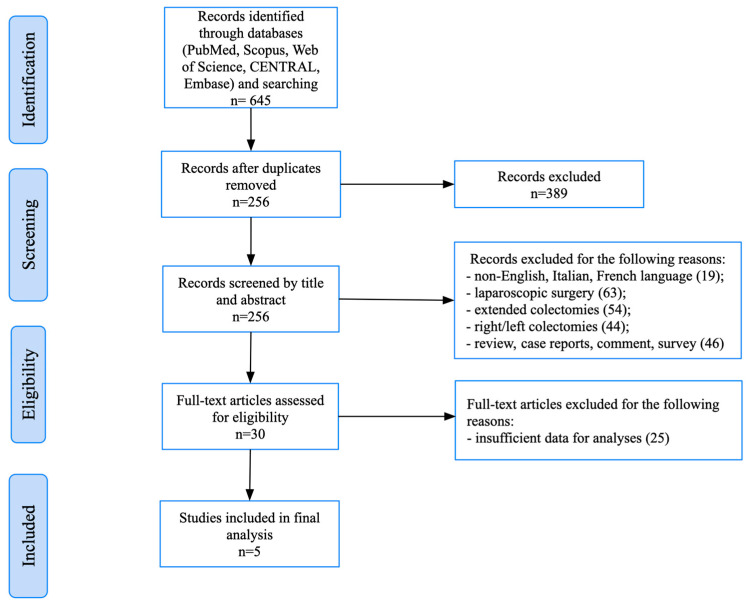
PRISMA flowchart.

This systematic review was conducted in accordance with the Preferred Reporting Items for Systematic Reviews and Meta-Analyses (PRISMA) guidelines [8] (Figure 1). The study protocol was developed a priori and registered in the PROSPERO database (Registration ID: CRD420251119736).

### 2.1. Search Strategy

A comprehensive literature search was performed using the electronic databases PubMed (MEDLINE), Embase, Scopus, Web of Science, and the Cochrane Central Register of Controlled Trials (CENTRAL), from their inception to August 2025. The search strategy combined terms related to robotic surgery, splenic flexure, transverse colon, segmental colectomy, and colorectal malignancy, with database-specific syntax adjustments applied. The reference lists of relevant reviews were also screened to identify additional studies, although these sources were not included in the final analysis.

### 2.2. Eligibility Criteria

Studies were considered eligible if they specifically reported on robotic segmental colectomy for malignant tumors of the splenic flexure or mid-transverse colon, and if they provided sufficient surgical and perioperative outcomes. We excluded single case reports and 2 case technical notes to avoid studies involving sparse, non-standardized reporting and to ensure minimally interpretable event rates (e.g., morbidity, anastomotic leakage). A ≥3 case threshold also improved the comparability across cohorts within our descriptive, single-arm framework and in regard to the subsequent GRADE appraisal.

Publications in English, French, or Italian were accepted. Conversely, case reports involving fewer than three patients, studies limited to right or left colectomies, or case series describing non-robotic or hybrid procedures without any clearly distinguishable robotic data were excluded. Reviews, conference abstracts without full text, editorials, and letters were also not considered. Primary endpoints were the conversion rate, postoperative morbidity, anastomotic leakage, and mortality, while secondary endpoints included operative variables, recovery outcomes, and oncological adequacy, assessed according to the R0 resection, lymph node yield, and recurrence rate.

### 2.3. Study Selection and Data Extraction

The selection process was conducted independently by two reviewers (A.M. and S.G.), who first screened the titles and abstracts and, subsequently, assessed the full text of potentially relevant articles. Any disagreements were resolved through discussions, with recourse to a third reviewer when necessary (G.L.). For each study, the data were systematically extracted using a predefined template. The variables collected included the study characteristics, patient demographics, and tumor location, surgical details, such as the type of resection performed, operative time, blood loss, and conversion rate, as well as information on the anastomotic technique used (manual versus mechanical, intracorporeal versus extracorporeal). Postoperative outcomes were also recorded. Oncological parameters, such as the lymph node yield, R0 resection rate, and recurrence rate, when available, were noted.

The results were summarized in structured tables, and pooled descriptive statistics were calculated when possible, using SPSS software, version 12.0 (SPSS Inc., Chicago, IL, USA). Given the heterogeneity of the study design and reporting, no formal meta-analysis was performed. Instead, weighted and unweighted pooled values were presented for the most relevant perioperative and oncological endpoints. Forest plots and tests for interaction were not conducted because the included studies reported single-arm outcomes, without consistent comparator arms or extractable effect sizes. Accordingly, this review provides a descriptive, non-comparative synthesis of the results, rather than a meta-analysis.

### 2.4. Risk of Bias Assessment

Due to the anticipated predominance of non-randomized and retrospective studies, the methodological quality and risk of bias were assessed using the Newcastle–Ottawa Scale (NOS) and the Risk of Bias in Non-randomized Studies of Interventions (ROBINS-I) [9,10]. Each study was evaluated independently by two authors.

### 2.5. Certainty of Evidence (GRADE)

We rated the certainty for each key outcome (conversion, overall morbidity, anastomotic leakage, R0 resection, and ≥12 lymph nodes) using the GRADE framework. This method considers five domains: risk of bias, inconsistency, indirectness, imprecision, and publication bias. Because the evidence base was small, retrospective, and heterogeneously reported, we anticipated concerns regarding the risk of bias, inconsistency, and imprecision. To avoid an overly conservative, blanket downgrading approach in this single-arm surgical context, we applied a pragmatic adaptation: objective oncologic endpoints with consistent reporting (R0, ≥12 nodes) were rated as *moderate*, whereas perioperative outcomes (conversion, morbidity, leakage) were rated as *low*, due to sparse data and reporting variability.

## 3. Results

### 3.1. Study Selection

The initial database search yielded a total of 645 records. After the removal of duplicates and screening of the titles and abstracts, 30 full-text articles were assessed for eligibility. Of these, five retrospective studies met the predefined inclusion criteria and were included in the final analysis [11,12,13,14,15]. A PRISMA flow diagram illustrating the study selection process is provided in Figure 1.

### 3.2. Risk of Bias Assessment

Table 1 provides a comprehensive overview of the risk of bias evaluation across the included studies. The methodological quality of the included studies was generally high, despite their retrospective design. The NOS scores ranged from 7 to 8, corresponding to high quality in regard to the comparative cohorts and the single-surgeon series [11,12,13,14]. Jung’s case series could not be assessed with the NOS and was considered at serious risk of bias, according to the ROBINS-I tool, due to the very small sample size and the lack of a control group [15]. Two studies were rated as having a moderate risk of bias, while the remainder were judged to be at low risk. The main concerns were residual confounding, retrospective data collection, incomplete perioperative information, and the limited generalizability of single-surgeon series.

Overall, the strength of the evidence can be considered moderate, constrained by the retrospective design, small patient cohorts, and potential selection bias. Consequently, the conclusions should be interpreted with caution.

### 3.3. Study Characteristics

The five included studies were all retrospective in design and collectively reported on 74 patients undergoing robotic resection for malignancies located at the splenic flexure or mid-transverse colon. The studies were conducted in four countries: Italy (n = 2), France, China, and South Korea. Three were comparative case series (two with propensity score matching) and two were single-surgeon case series. All the patients underwent a robotic segmental colectomy, with three studies including comparative laparoscopic cohorts. The robotic platforms utilized included the da Vinci S, Si, and Xi System^®^ (Intuitive, Sunnyvale, CA, USA).

A detailed overview of the study characteristics is provided in Table 2.

### 3.4. Patient and Tumor Characteristics

The patient demographics were variably reported. Patients were generally in the sixth to seventh decade of life, with a slight male predominance across the cohorts (overall distribution approximating 2:1). The American Society of Anesthesiologists (ASA) status, when available, indicated that most patients were classified as I–II. The tumor location was the splenic flexure in 31 patients (41.9%) and the mid-transverse colon in 43 patients (58.1%).

### 3.5. Surgical Technique and Intraoperative Outcomes

The technical details and intraoperative results are summarized in Table 3. All the procedures were performed using a robotic approach, although the extent of the robotic use varied depending on the anastomotic strategy. In the resections of the mid-transverse colon, vascular control invariably involved ligation of the middle colic vessels at their origin [11,12,14]. For splenic flexure cancers, the vascular strategies were more heterogeneous: Zhang et al. described artery-guided dissection, with division of the inferior mesenteric vein and left colic artery, while Monsellato et al. reported on a lymphadenectomy of the inferior mesenteric vein, inferior mesenteric artery, left colic artery, and both the main trunk and left branch of the middle colic vessels, while preserving the IMV [13,15]. Anastomotic reconstruction also varied: two studies performed extracorporeal stapled side-to-side anastomoses, one reported an intracorporeal hand-sewn end-to-end technique, and one described an intracorporeal stapled side-to-side approach. In the multicenter study by Milone et al., both intra- and extracorporeal anastomoses were performed, but the specific distribution among the 18 robotic cases was not reported, as the analysis was pooled with the laparoscopic group [14]. The use of indocyanine green (ICG) fluorescence was inconsistently reported. The operative time ranged from 157.5 to 267 min. Blood loss was consistently low (~100 mL). Conversion to open surgery was reported exclusively by Monsellato et al. (3 of 12 patients, 25%) [15].

### 3.6. Postoperative Outcomes and GRADE Certainty

The length of stay was typically 7–9 days. Across the five case series (n = 74), postoperative morbidity was 16.2% (12/74; study range 0–33.3%) and anastomotic leakage occurred in 5.4% cases (4/74; 0–11.1%). Thirty-day mortality was 0; reoperation occurred in 1/74 (1.4%) cases; one patient developed severe pulmonary failure. Comparator data were inconsistent or absent, so the relative effects could not be estimated. Using a pragmatic GRADE approach for single-arm surgical data, the certainty for these perioperative endpoints (conversion rate, morbidity) was rated low due to the small retrospective cohorts involved, reporting heterogeneity, and the occurrence of only a few events. The study-level outcomes are reported in Table 4, and the pooled absolute risks, along with the study ranges and GRADE certainty, are summarized in Table 5.

### 3.7. Oncological Outcomes

R0 resection was achieved in all 74 cases (100%). The tumor size was generally 3–5 cm. The lymph node yield varied across the case series, ranging from 6 to 8 nodes in the small single-surgeon report by Jung et al., to a mean of approximately 16–20 nodes in larger comparative case series, exceeding the oncological minimum of 12 in most patients. The resection margins were consistently negative (R0). The follow-up duration was heterogeneous, ranging from early postoperative reporting to up to 72 months. When reported, long-term oncologic outcomes were favorable: no recurrence in the cohorts described by De Angelis, Zhang, or Jung, and one recurrence with synchronous liver and lung metastases in the study by Monsellato (per-study details in Table 4).

### 3.8. Descriptive Stratifications

The outcomes according to the tumor site, anastomotic technique, and center type are summarized in Table 6. Mid-transverse cases (n = 43) showed higher morbidity (20.9%) and leakage rates (7.0%) compared with splenic flexure cases (n = 31), which had lower morbidity (9.7%) and leakage rates (3.2%), but a higher conversion rate (9.7%, all from one single-surgeon case series). Extracorporeal stapled anastomoses (n = 41) had a morbidity of 12.2% and a leakage rate of 4.9% with no conversions, whereas intracorporeal anastomoses (n = 15) had lower morbidity (6.7%), no leaks, but higher conversions (20%, single-surgeon case series). The multicenter cohort (n = 18) showed higher morbidity (33.3%) and a higher leakage rate (11.1%) compared with pooled single-center cohorts (n = 56; morbidity 10.7%, leakage 3.6%, conversion 5.4%). Platform generation and ICG use were inconsistently reported and, thus, could not be analyzed. Subgroup effect sizes were not extractable, and no interaction tests were performed.

## 4. Discussion

### 4.1. Technical Challenges and Role of Robotic Assistance

The splenic flexure and mid-transverse colon represent some of the most technically demanding regions for colorectal surgery, due to their variable vascular anatomy, the need for dual lymphatic drainage, and the close proximity to the spleen, pancreas, and stomach [16,17]. The oncological adequacy of segmental resection in this region has long been debated, with many surgeons historically favoring extended colectomy to ensure comprehensive lymphadenectomy [18,19,20]. Nevertheless, recent evidence suggests that a more conservative approach may be equally effective. For transverse colon cancers, a systematic review and meta-analysis by Morarasu et al. demonstrated no significant differences in overall or disease-free survival between patients undergoing segmental and extended colectomies [21]. Segmental resections were further associated with a shorter operative time, reduced intraoperative blood loss, and preservation of the bowel length, while still achieving adequate lymph node harvest and R0 resection rates. Similarly, an analysis of the National Cancer Database (NCDB), involving more than 60,000 patients, confirmed that segmental colectomy offers survival outcomes comparable to extended resections [22]. For splenic flexure cancers, additional large-scale and multicenter study data have reported equivalent oncological results between segmental and extended colectomies, reinforcing the oncological adequacy of a limited resection in this region [23,24]. Collectively, these findings strengthen the rationale for using segmental resection as an oncologically sound strategy.

Prior studies have shown that laparoscopy can match open surgery, even in complex oncologic settings. Robotics is a natural evolution of this minimally invasive pathway [25,26]. The platform’s three-dimensional vision, wristed instruments, and stable ergonomics enable precise plane-based dissection and reliable control of the left colic and the left branch of the middle colic arteries [27]. Compared with laparoscopy, it reduces the risk of splenic or pancreatic injury and facilitates medial-to-lateral dissection, flexure takedown, and the identification of key pedicles, particularly in obese patients, with a thick abdominal wall and a short transverse mesocolon.

### 4.2. Anastomotic Strategies

Anastomotic reconstruction varied across the case series, underscoring the lack of a standardized approach in this region. Two studies reported extracorporeal stapled side-to-side anastomoses, one an intracorporeal hand-sewn end-to-end technique, and another, an intracorporeal stapled approach. In the multicenter case series by Milone et al., both intra- and extracorporeal methods were used, but the robotic subgroup was not stratified, because the analyses were pooled with laparoscopy [14]. This heterogeneity likely mirrors differences in surgical experience and institutional preference, rather than a proven superiority of one method over another.

The limited use of intracorporeal anastomosis is notable given that the robotic platform facilitates stable exposure, reduced mesenteric traction, and smaller extraction sites. Moreover, being performed entirely within the abdominal cavity, intracorporeal reconstruction spares the surgeon from having to perform the extensive mobilization otherwise required to exteriorize the colonic stumps when using extracorporeal techniques [28,29,30]. This advantage is exemplified by Jung et al., who reported on the feasibility of intracorporeal hand-sewn end-to-end anastomoses, without the need for undue colonic mobilization [12].

The analysis performed by Milone et al. represents an excellent example of the current evidence and illustrates well the points discussed above: in a propensity matched comparison of 33 intracorporeal versus 33 extracorporeal anastomoses, intracorporeal reconstruction was associated with a shorter operative time, faster recovery of the bowel function, and a markedly lower complication burden, including anastomotic leaks, compared with extracorporeal reconstruction, while the long-term oncologic outcomes remained equivalent [14].

These findings are consistent with evidence from right colectomies, such as the prospective ANCOR trial, where intracorporeal reconstruction similarly reduced complications and accelerated postoperative recovery, despite modestly longer operative times [31].

Overall, intracorporeal anastomosis appears to be a valid reconstructive option in robotic segmental colectomy, although its adoption remains inconsistent, likely reflecting variability in the relevant expertise and the absence of standardized protocols. Fluorescence angiography was not routinely reported. This support tool, already used in multiple laparoscopic contexts, is now incorporated into robotic systems (Firefly^®^ mode), providing additional intraoperative guidance and potentially improving perfusion assessment [32,33,34].

### 4.3. Operative and Perioperative Outcomes

Across the pooled analysis of the five studies, the advantages of robotics were associated with consistent operative and perioperative results. The operative time was longer than in standard colectomies, reflecting the anatomical complexity rather than platform limitations. Conversion to open surgery was required in only 4.1% of patients, indicating stable procedural control, even in regard to anatomically demanding dissections. The morbidity (16.2%) and leakage rates (5.4%) were within expected colorectal benchmarks, indicating that robotic surgery does not carry an additional perioperative burden in this context. Importantly, mortality was zero and reoperation rare. R0 resection was universal, and the mean lymph node yield exceeded oncologic standards. Hospital stays ranged between 6 and 8.6 days, confirming that postoperative recovery is not negatively impacted, despite the demanding nature of the procedure.

This uniform adequacy, even across small and heterogeneous case series, highlights the ability of the robotic technique to ensure reproducible short-term outcomes and oncological reliability.

### 4.4. Operator Experience and Learning Curve

The concept of the learning curve has been widely investigated across different surgical fields and has been shown to vary substantially depending on the procedure and approach adopted. Different operative modalities (open, laparoscopic, robotic) are associated with distinct learning patterns, underscoring the multifactorial nature of surgical proficiency [35]. In colorectal surgery, robotics may shorten and standardize the learning curve through enhanced visualization, stable ergonomics, and wrist-based instrumentation [36,37,38]. Several of the reports included in this analysis are single-surgeon case series, which limits the generalizability of the findings and likely influences the technical outcomes. Conversions were clustered into one single-surgeon cohort (3/12; 25%), whereas the remaining case series reported 0% conversion; conversely, anastomotic leaks were absent in the single-surgeon cohorts (0/15), but occurred at a low frequency in the multi-operator comparative cohort. The operative time varied widely (≈157.5–268 min), consistent with differences in the case selection and stage along the learning curve. Although we could not formally model the learning curves (e.g., CUSUM) due to the small sample size and retrospective design of the studies included, future studies should report on the surgeon-level volume, sequence number, and proctoring to enable learning curve-adjusted benchmarking of the outcomes, such as conversion and leakage rates.

### 4.5. Cost-Effectiveness Considerations

The economic profile of robotic segmental colectomy likely varies by center and case mix. Capital and maintenance costs, instrument utilization, and case volume (learning curve and amortization effects) must be weighed against the potential gains, such as lower conversion, improved ergonomics, a comparable length of stay, and team-level efficiency [39,40]. Given the single-arm nature and small sample size in the included studies, comparative cost effectiveness remains uncertain. We advocate standardized cost reporting (fixed vs. variable costs, instrument counts, operative time, length of hospital stay, readmissions) and prospective, adequately powered comparative studies to determine whether the clinical benefits translate into net economic value across different healthcare settings.

### 4.6. Strengths

To our knowledge, this is the first focused synthesis of robotic segmental resections for splenic flexure and mid-transverse colon malignancies, which are two anatomically demanding locations. Beyond cataloguing technical variants (anastomotic strategy, vascular approach), this review shows consistent oncologic adequacy across the case series, with universal R0 reporting and a high proportion of patients meeting the ≥12 lymph node benchmark. These outcomes improve the interpretability of such data and may help to guide technical decisions in this context.

### 4.7. Limitations

The available evidence consists of retrospective, small cohorts, several of which are single-surgeon case series, with incomplete reporting of key modifiers (robot platform generation, ICG use), and substantial heterogeneity in regard to the case mix and technique used (tumor site, anastomosis type, platform). This precluded formal subgroup meta-analyses and tests for interactions. The follow-up period was short or variably reported, limiting long-term oncologic inferences. In regard to the NOS and ROBINS-I, the overall methodological quality was acceptable (comparative cohorts were generally higher; single-surgeon case series were at greater risk of bias). Using this pragmatic GRADE adaptation, the certainty ranged from low (perioperative endpoints) to moderate (objective oncologic endpoints). In conclusion, the results remain hypothesis generating and merit confirmation using larger, prospective multicenter studies.

### 4.8. Future Directions

Future research should aim to consolidate the role of the robotic approach in segmental colectomy for the treatment of splenic flexure and mid-transverse colon cancers through the use of prospective multicenter studies, with standardized protocols. Key areas for further investigation include the impact of the learning curve on surgical performance, the reproducibility of intracorporeal anastomosis as a reconstructive strategy, and the oncological validation of segmental colectomy as a safe alternative to extended resections.

## 5. Conclusions

Robotic segmental colectomy for the treatment of splenic flexure and mid-transverse colon cancers appears feasible and safe, with acceptable short-term and oncologic outcomes, and it can be used to facilitate complex dissection and intracorporeal reconstruction. Yet, the current evidence is limited to small retrospective case series. Multicenter prospective studies are needed to confirm the oncologic safety, define the learning curves, and determine its cost effectiveness before broad adoption.

## Figures and Tables

**Table 1:** Risk of bias assessment.

Author (Year)	Country	Study Design	NOS Score	NOS Quality	ROBINS-I Overall Risk	Key Sources of Bias
De Angelis et al., 2015 [11]	France	Comparative retrospective	8	High	Moderate	Retrospective design with potential unmeasured confounders; no PSM.
Jung et al., 2015 [12]	South Korea	Single-surgeon retrospective series	NA	NA	Serious	Lack of control group and small sample size; retrospective design.
Zhang et al., 2021 [13]	China	Comparative (PSM) retrospective	8	High	Low	Use of PSM reduced imbalance; low risk from residual confounding.
Milone et al., 2022 [14]	Italy	Comparative (PSM) retrospective	8	High	Low	Matching reduced confounding, but residual bias from selection and retrospective data collection.
Monsellato et al., 2024 [15]	Italy	Single-surgeon retrospective series	7	High	Moderate	Single-operator experience limits generalizability; small sample size; moderate risk from selective reporting.

Risk of bias and methodological quality of the included studies, evaluated using the Newcastle–Ottawa Scale (NOS) and the Risk of Bias in Non-randomized Studies of Interventions (ROBINS-I). NOS scores range from 0 (lowest) to 9 (highest quality), categorized as low (≤3), moderate (4–6), or high (7–9). According to the ROBINS-I methodology, the global assessment of risk of bias reflects the highest level observed among the seven domains considered (low, moderate, or serious). PSM, propensity score matching; NA, not available.

**Table 2:** Study and patient characteristics.

Author (Year)	Patients (n)	Age (Years)	Gender (M/F)	BMI (kg/m^2^)	ASA I–II	ASA III–IV	Tumor Location
De Angelis et al., 2015 [11]	22	72.2 *	15/7	24.1 *	22	0	Mid-Transverse Colon
Jung et al., 2015 [12]	3	59.6 *	2/1	26.1 *	NA	NA	Mid-Transverse Colon
Zhang et al., 2021 [13]	19	58 **	12/7	23.5 **	19	0	Splenic Flexure
Milone et al., 2022 [14]	18	74.2 **	15/3	25 **	NA	NA	Mid-Transverse Colon
Monsellato et al., 2024 [15]	12	70 **	6/6	27.4 **	10	2	Splenic Flexure

Summary of included studies reporting on robotic segmental colectomy for splenic flexure and mid-transverse colon cancers. Variables include patient demographics, BMI (body mass index), ASA (American Society of Anesthesiologists) status, and tumor location; * mean; ** median.

**Table 3:** Reported pre- and intraoperative data.

Author (Year)	Robotic Platform	Vessel Ligation	Anastomotic Technique	IOBL (mL)	OT (min)	Intraoperative Complications (n, %)	Conversion Rate (n, %)
De Angelis et al., 2015 [11]	da Vinci Surgical System	MCA, MCV	Extracorporeal, side-to-side, mechanic	100	267.95 *	0 (0%)	0 (0%)
Jung et al., 2015 [12]	da Vinci S Surgical System	MCA, MCV	Intracorporeal, end-to-end, hand-sewn	NA	268.3 *	0 (0%)	0 (0%)
Zhang et al., 2021 [13]	da Vinci Si Surgical System	IMV, LCA	Extracorporeal, side-to-side, mechanic	100	170 **	0 (0%)	0 (0%)
Milone et al., 2022 [14]	NA	MCA, MCV	NA	NA	157.5 **	0 (0%)	0 (0%)
Monsellato et al., 2024 [15]	da Vinci Si/Xi Surgical System	LCV, LCA, LMCA, LMCV	Intracorporeal, side-to-side, mechanic	NA	267 **	0 (0%)	3 (25%)

Pre- and intraoperative findings from the included studies, detailing the robotic platforms employed, the vascular ligation strategies, the type of anastomosis performed, and operative metrics, such as blood loss, operative time, intraoperative complications, and conversion to open surgery. IOBL = intraoperative blood loss; OT = operative time; MCA = middle colic artery; MCV = middle colic vein; IMV = inferior mesenteric vein; LCA = left colic artery; LCV = left colic vein; LMCA = left branch of MCA; LMCV = left branch of MCV; NA = not available; * mean; ** median.

**Table 4:** Reported postoperative data.

Author (Year)	LOS (Days)	Morbidity (n, %)	Complication Type	Anastomotic Leak	30-Day Mortality (n,%)	Tumor Size (cm)	Resection Margin	Nodes (n)	Follow-Up (Months)
De Angelis et al., 2015 [11]	7 *	3 (13.6%)	1 leak; 1 ileus; 1 wound infection	1	0 (0%)	4.7 *	R0	17.5 *	2 *
Jung et al., 2015 [12]	8.6 *	0 (0%)	-	0	0 (0%)	3.2 *	R0	7 *	72 *
Zhang et al., 2021 [13]	8 **	2 (10.5%)	1 leak; 1 pulmonary infection	1	0 (0%)	5 **	R0	18 **	24 **
Milone et al., 2022 [14]	8 **	6 (33.3%)	2 leaks; 2 wound infections; 2 bleeds	2	0 (0%)	NA	R0	14.5 **	3.4 **
Monsellato et al., 2024 [15]	6 **	1 (8%)	1 pulmonary failure	0	0 (0%)	NA	R0	20 **	42 **

Postoperative results across the included case series, including the length of stay, overall morbidity, type of complications, anastomotic leaks, mortality, tumor size, resection margins, lymph node yield, and follow-up duration. LOS, length of stay; NA, not available; * mean; ** median.

**Table 5:** **Summary of Findings for Robotic Segmental Resection with GRADE Certainty.** Aggregated absolute risks across five retrospective robotic series (n = 74), shown with study ranges and GRADE certainty. These are single-arm descriptive estimates (not comparative).

Outcome	Pooled n/N (%)	Study Range	Studies (n)	Certainty (GRADE)	Brief Reasons for Rating
Open Conversion	3/74 (4.1%)	0–25%	5	LOW	Observational single-arm evidence; small samples and few events → imprecision.
Overall Morbidity	12/74 (16.2%)	0–33.3%	5	LOW	Observational design; heterogeneous reporting; downgraded for risk of bias/imprecision.
Anastomotic Leak	4/74 (5.4%)	0–11.1%	5	LOW	Observational design; small samples → imprecision; objective ascertainment.
R0 Resections	74/74 (100%)	100% in all	5	MODERATE	Objective oncologic endpoint; consistent across studies; acceptable precision.
≥12 Lymph Nodes	71/74 (95.9%)	≈89–100%	5	MODERATE	Objective endpoint; high and consistent attainment; acceptable precision.

Aggregated data from across all of the included studies, reporting on the patient demographics, tumor location, anastomotic techniques, operative time, conversion rate, postoperative outcomes, 30-day mortality, lymph node yield, and R0 resection rates. Reported values reflect the original format (mean, median, or absolute values). Because of the heterogeneous reporting across the studies, pooled summary values should be interpreted with caution and are presented here descriptively rather than as formal meta-analysis estimates. BMI, body mass index; NA, not available; LOS, length of stay.

**Table 6:** **Descriptive stratifications for robotic segmental resection.** Outcomes stratified by tumor site, anastomotic technique, and center type across five retrospective robotic series (n = 74). Results are presented as raw n/N (%) event rates. These are single-arm descriptive data, not comparative estimates. Platform generation and ICG use were inconsistently reported and therefore could not be analyzed. These data are descriptive only, with subgroup effect sizes not extractable. Therefore, no formal interaction testing was performed.

Stratification	Subgroup (n)	Morbidity n/N (%)	Anastomotic Leak n/N (%)	Conversion n/N (%)
Tumor Site	Mid-transverse (n = 43)	9/43 (20.9%)	3/43 (7.0%)	0/43 (0%)
	Splenic flexure (n = 31)	3/31 (9.7%)	1/31 (3.2%)	3/31 (9.7%)
Anastomosis	Extracorporeal (n = 41)	5/41 (12.2%)	2/41 (4.9%)	0/41 (0%)
	Intracorporeal (n = 15)	1/15 (6.7%)	0/15 (0%)	3/15 (20%)
Center Type	Multicenter cohort (n = 18)	6/18 (33.3%)	2/18 (11.1%)	0/18 (0%)
	Single Center pooled (n = 56)	6/56 (10.7%)	2/56 (3.6%)	3/56 (5.4%)

## Data Availability

All data presented in this study have been extracted from the literature.
